# Etiology of asymptomatic bacteriuria, antimicrobial susceptibility patterns and associated risk factors among pregnant women attending antenatal clinic in western Kenya

**DOI:** 10.1371/journal.pgph.0004347

**Published:** 2025-03-20

**Authors:** Dorothy Atieno Odindo, Benjamin Ochieng, Fredrick Onduru, Caroline Ouma, Daniel Onguru, Shehu Shagari Awandu

**Affiliations:** 1 Department of Biomedical Sciences, Jaramogi Oginga Odinga University of Science and Technology, Bondo, Kenya; 2 Center for Global Health Research, Kenya Medical Research Institute, Kisumu, Kenya; 3 Department of Biological Sciences, Jaramogi Oginga Odinga University of Science and Technology, Bondo, Kenya; Universiti Kebangsaan Malaysia, MALAYSIA

## Abstract

Approximately 11.1% of pregnant women in Africa experience asymptomatic bacteriuria (ASB), and its proper understanding is critical due to its risks, including pyelonephritis in mothers and fetal mortality. However, a significant gap remains in understanding the optimal screening and treatment protocols for ASB in pregnant women. We assessed the etiology of asymptomatic bacteriuria, antimicrobial susceptibility patterns, and associated risk factors among pregnant women attending an antenatal clinic in western Kenya. Using a cross-sectional quantitative design, 285 asymptomatic pregnant women were recruited, interviewed using a questionnaire and provided urine for culture. Microbial susceptibility was tested using the Kirby Bauer disk diffusion technique and interpreted based on the Clinical and Laboratory Standards Institute guidelines. Asymptomatic bacteriuria prevalence was 16.3% (44/270), and increased with gestational age for trimester 1, 2 and 3 at 12%, 17.7% and 19.3%, respectively. Of the ASB cases, 45.5% (20/44) were caused by gram-negative bacteria, and 56.8% (25/44) by gram-positive bacteria. Isolated gram-negative bacteria were *Escherichia coli* (80%), *Klebsiella pneumoniae* (10%), *Proteus mirabilis* (5%) and *Pseudomonas aeruginosa* (5%), while the isolated gram-positive bacteria were coagulase-negative *Staphylococcus* species (52%), *Enterococcus* species (20%), *Staphylococcus aureus* (16%) and *Streptococcus agalactiae* (8%). Antibiotics with high sensitivity by gram-negative bacteria were azithromycin, meropenem, and tobramycin (100% susceptibility), while all isolates (100%) were resistant to trimethoprim-sulfamethoxazole. Gram-positive isolates were highly (100%) sensitive to gentamicin, ofloxacin, clindamycin and ampicillin, and 56% were resistant to trimethoprim-sulfamethoxazole. Women with at least a secondary school education had 2.47 times higher odds of getting asymptomatic bacteriuria (AOR = 2.47, 95% CI [1.09, 5.98], p = 0.036), while women between 25-34 years of age were at 2.23 times higher odds of ASB (AOR = 2.23, 95% CI [1.07, 4.63], p = 0.030). There is a need for extensive antimicrobial susceptibility testing to identify safe and effective antibiotics for treating ASB.

## Introduction

Globally, asymptomatic bacteriuria (ASB), defined as the presence of bacteria in a urine sample without any indication of a urinary tract infection [1] frequently affects both genders. However, women are more prone to it especially as they age [2,3]. While asymptomatic bacteriuria itself is not typically harmful, it can increase the risk of developing more serious infections such as cystitis, pyelonephritis and pyonephrosis [4].

There is a considerable difference in the prevalence of asymptomatic bacteriuria in pregnancy among different population settings. Asymptomatic bacteriuria accounts for 70% of all infections related to urinary tract infection and between 2 and 15% of pregnant women will have ASB during pregnancy [5]. While certain Asian countries have reported higher percentages of 17% to 25.3%, studies have also found that rates in industrialized nations range from 2% to 10% [6,7]. Research in Nigeria reported a prevalence of ASB among expectant mothers ranging between 15.1% to 86.6% [2], Ghana reported a 5% prevalence, north and south Ethiopia reported a 21.1% and 18.8% prevalence rates respectively [8]. A study conducted in Kenya reported a 21.5% prevalence [9].

Single bacterium presence is responsible for a significant proportion of urinary tract infections in pregnant women. However, several bacteria, particularly those with increased antibiotic resistance due to gene alterations can be implicated in complex urinary tract infections (UTI) cases [2]. *Escherichia coli* is the most commonly found microorganism in ASB and accounts for between 70-90% of ASB related UTI [10,11]. Other microbial agents such as *Streptococcus agalactiae, Pseudomonas aeruginosa, Proteus mirabilis, Klebsiella pneumonia, Staphylococcus. saprophyticus, Staphylococcus aureus and Enterococcus faecalis* are also involved [12].

Research has shown that pathogenic bacteria, particularly uropathogens, are quickly developing antibiotic resistance, with multidrug-resistant strains becoming significant threats [8]. Pregnancy associated factors such as increased glucose levels in urine (affecting 70% of pregnant women),low immunity during pregnancy and the anatomical proximity of the anal canal to the urethra, create favorable conditions for bacterial growth in the urinary tract [13].

Additional risk factors include maternal age, lower socioeconomic status, a history of urinary tract infections, anemia, catheterization, multiparity, aminoaciduria, and diabetes mellitus [1,10]. Untreated bacteriuria infection during pregnancy can lead to complications such as premature births, low birth weight, preterm labor, intrauterine growth retardation, fetal death, and increased prenatal mortality and morbidity. Therefore routine screening for asymptomatic infections should be a public health priority and researchers recommend routine culture screening for all pregnant women who visit prenatal clinics [4,13].

Information on causative bacteria, antimicrobial susceptibility pattern, and ASB prevalence is limited, despite its importance for maternal to child health. The prevalence of asymptomatic bacteriuria in pregnancy remains largely unknown across most regions in sub-Saharan Africa, with no published data specifically from western Kenya. A study conducted in Nairobi highlighted the need for further research and random sampling in other regions of Kenya to gain a true understanding of bacteriuria infections among pregnant women [9].

The socioeconomic environments in different regions across Kenya lead to unique predisposing factors for infections. Consequently, data on bacteriuria infections from both urban and rural settings is necessary. This approach helps determine if regional differences influence the prevalence and risk factors associated with bacteriuria in pregnant women [9]. Available data on antimicrobial susceptibility is insufficient due to the limited number of studies focusing on asymptomatic bacteriuria in pregnant women. In addition, the existing information is inadequate for guiding healthcare practices for pregnant women with ASB-related complications.

Here, we conducted comprehensive investigations to access the etiology of asymptomatic bacteriuria, antimicrobial susceptibility patterns and associated risk factors among women attending antenatal clinic in western Kenya. This study findings will help policy makers and healthcare providers in western Kenya to standardize antenatal care practices, including mandatory ASB screening to ensure timely identification and treatment of ASB to reduce complications such as pyelonephritis, low birth weight and fetal mortality. The findings will also guide the implementation of targeted treatment interventions hence reducing antimicrobial resistance among bacterial pathogens.

## Materials and methods

### Study design, aim and setting

We carried out a cross sectional study to determine the etiology of asymptomatic bacteriuria, antimicrobial susceptibility patterns of the isolated pathogens and associated risk factors (maternal age and educational level) among pregnant women attending an antenatal clinic at Chulaimbo Sub county hospital, in western Kenya. The study area is predominantly rural, with most residents of low socioeconomic status, which increases their exposure to infectious agents. Additionally, the hospital serves as a referral center for dispensaries and health centers in the Sub-county and surrounding areas including both rural and urban populations, thus targeting a larger population.

The study targeted pregnant women during their routine antenatal care visits at Chulaimbo Sub -County Hospital. Pregnant women of all gestational periods who gave their written consent were included in the study. Pregnant women who had signs and symptoms of urinary tract infection, who were on antibiotic treatment or had used antibiotics in the previous two weeks when they came to the antenatal clinic and who did not give their written consent were excluded from the study.

### Sample size determination

(Cochran, 1998) formula was used in sample size determination where:


$${\text{n=(Z)}}^{\text{2}}\text{p}\;\text{q/}\;{\text{d}}^{\text{2}}$$

Where;

n = is the sample size

Z_=_ Z statistic for the level of confidence (for 95% confidence interval, Z value is 1.96).

p is the reported prevalence of ASB, 21.5% [9].

d is the precision/ margin of error, considered to be 0.05 to give good precision and smaller error of estimates.

Substituting:

= (1.96)^2^ 0.215(0.785)/0.05^2^

=259

The minimum calculated sample size using the Cochran formula above was 259. This sample size was adjusted by 10% to account for non-response.

Final sample size by 10% adjustment for non-response rate= (110*259)/100=284.9=285

### Specimen and data collection

A systematic sampling technique was used to recruit participants from 17^th^ January 2023 to 26^th^ September 2023. Based on the hospital’s previous performance reports, about 810 pregnant women were expected to visit the ANC during the period of study. This estimate was used to determine the sampling interval, by dividing the estimate into the sample size of 285 participants, the interval was 3. The first participant was selected randomly by use of a dice, then onward every third pregnant woman was invited to participate until the sample size was obtained. A written and signed informed consent was obtained from the participants and a written and signed assent obtained from the parents and caregivers of the under aged participants.

### Collection of clinical and demographic data

Demographic and clinical data such as age, socioeconomic status, occupation, gestational period, history of urinary tract infection were collected using a standardized questionnaire.

### Collection of urine for processing

One single void of fresh midstream urine (about 10ml) in a sterile leak-proof urine container was collected by each participant after receiving instructions on the collection procedure. The urine containers were disposable hence each participant had their own containers to avoid cross-contamination and further infection. The specimens were kept in a 2-8°C refrigerator and transported in a cooler box (2-8°C) within 2 hours of collection to the Kenya Medical Research Institute Bacteriology laboratory in Kisumu for analysis.

### Urine culture and bacterial identification

The urine samples were centrifuged at 10,000 rpm for 10 minutes and approximately 10µl inoculated onto Chocolate blood agar, Sheep blood agar and MacConkey agar media, (Becton Dickinson-BD). The inoculated agar plates were then incubated aerobically at 37°C for 18-24 hours. Bacterial isolates were identified by their colony characteristics, gram stain reaction, Coagulase test, Bile asculin test, Analytical profile index (API 20E),Optochin test,Christie Atkins Munch Petersen (CAMP) test and biochemical tests performed using standard laboratory methods of bacterial identification [14].

### Antimicrobial susceptibility testing

Antimicrobial test of the isolates was determined using the Kirby-Bauer disc diffusion and dilution methods [14] on Muller–Hinton agar (Oxoid Ltd, Hampshire, UK) against different antimicrobials depending on the organism isolated according to Clinical and Laboratory Standard Institute guidelines [15]. The following commercially prepared antibiotics were used chloramphenicol (30μg), trimethoprim-sulfamethoxazole (1.25μg/23.75μg), tetracycline (30μg), ciprofloxacin (5μg), nalidixic acid (30μg), ampicillin (10μg), gentamicin (10μg), azithromycin (15μg), meropenem (10μg), ceftriaxone (30 μg), amoxicillin-clavulanic acid (20/10 μg), tobramycin (10μg),oxacillin (1μg),ofloxacin (5μg) and clindamycin (2μg).

### Data analysis

Data generated/collected was categorized as case numbers and entered into a Microsoft Excel spreadsheet, and analysis performed using R software version 4.2.3. Descriptive statistics was used to determine the prevalence of asymptomatic bacteriuria, the proportion of bacteria, and the antimicrobial susceptibility profiles of bacteria. Bivariate and multivariable logistic regression determined factors associated with asymptomatic bacteriuria. The risk factors were assessed by odds ratios and confidence intervals. Variables with a *p*-value less than 0.20 in the bivariate analysis were selected for further analysis by multiple logistic regression. A *p*-value of less than 0.05 was taken as a cutoff point to determine variables that are independently associated with asymptomatic bacteriuria.

### Ethical considerations

Permission to carry out this study was obtained from the Board of Postgraduate Studies (BPS) of Jaramogi Oginga Odinga University of Science and Technology (JOOUST). Ethical approval was obtained from Jaramogi Oginga Odinga University of Science and Technology (JOOUST) research and ethical committee (Approval number: ERC 22/10/03-21). Permission to collect information was obtained from the National Commission for Science, Technology, and Innovation, (NACOSTI) (License number: NACOSTI/P/22/22366). Permission to carry out the study at the Sub-County Hospital was obtained from the County government of Kisumu department of health and sanitation (REF: GN133VOL.XII/ (484)). Clinical data of this study’s participants was obtained from the study participants, after a written and signed informed consent was obtained. A written and signed assent was obtained from the parents and caregivers of the underage participants. All participants were identified on a unique ID that links the samples with clinical and demographic information without revealing personal identifiers such as names to protect their anonymity and confidentiality.

## Results

**Fig 1:**
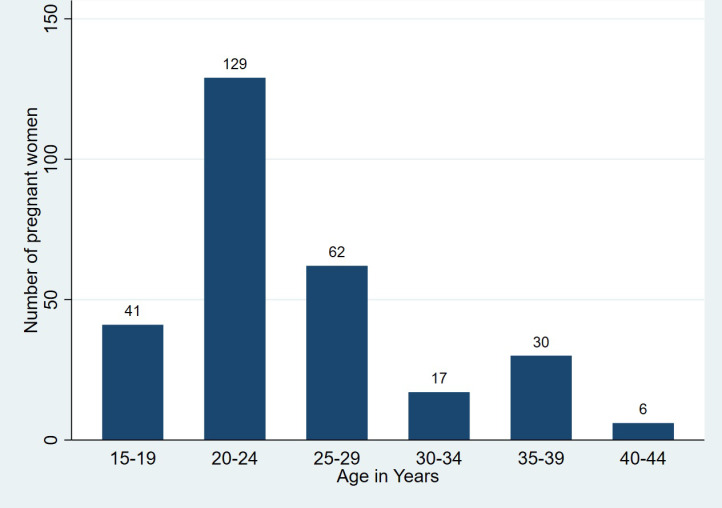
Age distribution of pregnant women attending antenatal clinics.

A total of 285 asymptomatic pregnant women were enrolled to participate in the study, 45.3% (129/285) of them were aged between 20 to 24 years old. 14.4% (41/285) of the enrolled women ranged between 15 to 19 years, while 2.1% (6/285) ranged between 40 to 44 years. The participants mean age was 26.6 years. The youngest enrolled participant was 15 years old while the oldest was 44 years old (Fig 1).

### Sociodemographic and clinical characteristics of pregnant women attending antenatal clinic in western Kenya

From the enrolled participants, almost half, 48.8% (139/285) were enrolled at trimester 2 of pregnancy, while 30.5% (87/285) and 20.7% (59/285) enrolled at trimester 1 and 3 respectively. About 58.9% (168/285) had previous history of urinary tract infection, 63.9% (182/285) were employed, and 66% (188/285) were married. Approximately, 69.8% (199/285) of the participants had attained secondary level of education (Table 1). About 86% (245/285) of the participants were using pit latrines and 63.5% (181/285) of the participants had two or more pregnancies. Most of the participants enrolled 59.6% (170/285) were in the age group of 15-24 years. Asymptomatic bacteriuria positivity was observed to increase by gestational age (trimester), from 12% (10/83) at trimester 1 to 17.7% (23/130) and 19.3% (11/57) for trimester 2 and 3 respectively. Similarly, asymptomatic bacteriuria positivity was high among women aged 25 years and above (24%, 18/75) as compared to those aged between 15-24 years (14.3%, 23/161) based on the row-wise proportions. None of the enrolled participants reported to have high blood pressure and diabetes (Table 1).

**Table 1:** Sociodemographic and clinical characteristics of pregnant women attending antenatal clinic in western Kenya.

Characteristic		Asymptomatic bacteriuria			
**N = 285** ^*1,2*^	**Overall, N = 270** ^***1***^	**Yes, N = 44** ^***1,3***^	**No, N = 226** ^***1,3***^	**p-value** ^*4*^
	n (%)	n (%)	n (%)	n (%)	
Urinary Tract Infection history	168 (58.9)	159 (100.0)	22 (13.8)	137 (86.2)	0.2
Occupation					0.14
*Unemployed/student*	103 (36.1)	94 (100.0)	11 (11.7)	83 (88.3)	
*Working/Employed*	182 (63.9)	176 (100.0)	33 (18.8)	143 (81.3)	
Marital status					0.5
*Single/divorced*	97 (34.0)	91 (100.0)	13 (14.3)	78 (85.7)	
*Married*	188 (66.0)	179 (100.0)	31 (17.3)	148 (82.7)	
Education level					0.2
*Primary and below*	86 (30.2)	78 (100.0)	9 (11.5)	69 (88.5)	
*Secondary*	140 (49.1)	136 (100.0)	28 (20.6)	108 (79.4)	
*Tertiary/university*	59 (20.7)	56 (100.0)	7 (12.5)	49 (87.5)	
Using Toilet	48 (16.8)	46 (100.0)	9 (19.6)	37 (80.4)	0.5
Using Pit latrine	245 (86.0)	232 (100.0)	37 (15.9)	195 (84.1)	0.7
Parity					>0.9
*First*	104 (36.5)	98 (100.0)	15 (15.3)	83 (84.7)	
*Second*	73 (25.6)	71 (100.0)	12 (16.9)	59 (83.1)	
*Third and above*	108 (37.9)	101 (100.0)	17 (16.8)	84 (83.2)	
Age (years)					0.077
*15-24*	170 (59.6)	161 (100.0)	23 (14.3)	138 (85.7)	
*25-34*	79 (27.7)	75 (100.0)	18 (24.0)	57 (76.0)	
*35-44*	36 (12.6)	34 (100.0)	3 (8.8)	31 (91.2)	
Trimester of pregnancy					0.4
*1st*	87 (30.5)	83 (100.0)	10 (12.0)	73 (88.0)	
*2nd*	139 (48.8)	130 (100.0)	23 (17.7)	107 (82.3)	
*3rd*	59 (20.7)	57 (100.0)	11 (19.3)	46 (80.7)	

^*1*^n (%) ^*2*^Column-wise proportion ^*3*^Row-wise proportion ^*4*^ Pearson’s Chi-squared test

### Pathogens identified and antimicrobial susceptibility pattern of gram-negative bacteria isolated from urine samples obtained from pregnant women

Almost half, 45.5% (20/44) of asymptomatic bacteriuria cases were gram-negative bacteria (Table 2). From these twenty cases of gram-negative bacteria, only four agents were identified; *Escherichia coli*, *Klebsiella pneumoniae, Proteus mirabilis* and *Pseudomonas aeruginosa*. Up to 80% (16/20) of gram-negative were *E. coli* and 10% (2/20) were *K. pneumoniae.* The remaining 5% each was accounted for by *P. mirabilis* (1/20) *and P. aeruginosa* (1/20)*.*

**Table 2:** Pathogens identified and antimicrobial susceptibility patterns of gram-negative bacteria isolated from urine samples obtained from pregnant women.

Characteristic	Overall, N = 20^*1*^ n (%)	*Escherichia coli*, N = 16^*1*^ n (%)	*Klebsiella pneumoniae*, N = 2^*1*^ n (%)	*Proteus mirabilis*, N = 1^*1*^ n (%)	*Pseudomonas aeruginosa*, N = 1^*1*^ n (%)
**Amoxicillin-clavulanic acid**					
*Intermediate*	2 (10)	0 (0)	2 (100)	0 (0)	0 (0)
*Resistant*	1 (5)	0 (0)	0 (0)	0 (0)	1 (100)
*Sensitive*	17 (85)	16 (100)	0 (0)	1 (100)	0 (0)
**Ampicillin**					
*Intermediate*	0 (0)	0 (0)	0 (0)	0 (0)	0 (0)
*Resistant*	18 (90)	16 (100)	0 (0)	1 (100)	1 (100)
*Sensitive*	2 (10)	0 (0)	2 (100)	0 (0)	0 (0)
**Azithromycin**					
*Intermediate*	0 (0)	0 (0)	0 (0)	0 (0)	0 (0)
*Resistant*	0 (0)	0 (0)	0 (0)	0 (0)	0 (0)
*Sensitive*	20 (100)	16 (100)	2 (100)	1 (100)	1 (100)
**Ceftriaxone**					
*Intermediate*	0 (0)	0 (0)	0 (0)	0 (0)	0 (0)
*Resistant*	1 (5)	0 (0)	0 (0)	0 (0)	1 (100)
*Sensitive*	19 (95)	16 (100)	2 (100)	1 (100)	0 (0)
**Chloramphenicol**					
*Intermediate*	0 (0)	0 (0)	0 (0)	0 (0)	0 (0)
*Resistant*	2 (10)	0 (0)	0 (0)	1 (100)	1 (100)
*Sensitive*	18 (90)	16 (100)	2 (100)	0 (0)	0 (0)
**Ciprofloxacin**					
*Intermediate*	1 (5)	0 (0)	0 (0)	0 (0)	1 (100)
*Resistant*	0 (0)	0 (0)	0 (0)	0 (0)	0 (0)
*Sensitive*	19 (95)	16 (100)	2 (100)	1 (100)	0 (0)
**Gentamicin**					
*Intermediate*	1 (5)	0 (0)	0 (0)	1 (100)	0 (0)
*Resistant*	0 (0)	0 (0)	0 (0)	0 (0)	0 (0)
*Sensitive*	19 (95)	16 (100)	2 (100)	0 (0)	1 (100)
**Meropenem**					
*Intermediate*	0 (0)	0 (0)	0 (0)	0 (0)	0 (0)
*Resistant*	0 (0)	0 (0)	0 (0)	0 (0)	0 (0)
*Sensitive*	20 (100)	16 (100)	2 (100)	1 (100)	1 (100)
**Nalidixic acid**					
*Intermediate*	0 (0)	0 (0)	0 (0)	0 (0)	0 (0)
*Resistant*	17 (85)	16 (100)	0 (0)	0 (0)	1 (100)
*Sensitive*	3 (15)	0 (0)	2 (100)	1 (100)	0 (0)
**Trimethoprim-sulfamethoxazole**					
Intermediate	0 (0)	0 (0)	0 (0)	0 (0)	0 (0)
Resistant	20 (100)	16 (100)	2 (100)	1 (100)	1 (100)
Sensitive	0 (0)	0 (0)	0 (0)	0 (0)	0 (0)
**Tetracycline**					
*Intermediate*	0 (0)	0 (0)	0 (0)	0 (0)	0 (0)
*Resistant*	4 (20)	0 (0)	2 (100)	1 (100)	1 (100)
*Sensitive*	16 (80)	16 (100)	0 (0)	0 (0)	0 (0)
**Tobramycin**					
*Intermediate*	0 (0)	0 (0)	0 (0)	0 (0)	0 (0)
*Resistant*	0 (0)	0 (0)	0 (0)	0 (0)	0 (0)
*Sensitive*	20 (100)	16 (100)	2 (100)	1 (100)	1 (100)

^1^n (%):- n ~ Frequency, (%) ~ Percentages

Generally, 85% (17/20) of these bacteria were sensitive to amoxicillin-clavulanic acid, these were 16 *E. coli* and 1 isolate of *P. mirabilis.* The vast majority*,* 90% (18/20) of bacteria were ampicillin resistant. These bacteria were 16, cases for *E. coli*, and a single case each for *P. mirabilis* and *P. aeruginosa*. All (100%; 20/20) of the four bacterial pathogens (*E. coli*, *K. pneumoniae*, *P. mirabilis* and *P. aeruginosa*) were sensitive to azithromycin, meropenem and tobramycin. Sensitivity to ceftriaxone, chloramphenicol, ciprofloxacin and gentamicin was observed among all *E. coli* (80%; 16/20) and *K. pneumoniae* (10%; 2/20) isolates. All *E. coli* (16/20) bacteria were resistant to nalidixic acid and all the gram-negative bacteria (20/20) were resistant to trimethoprim-sulfamethoxazole. *P. mirabilis* (5%; 1/20) showed resistance to ampicillin, chloramphenicol, gentamicin, tetracycline and trimethoprim-sulfamethoxazole. *P. aeruginosa* (5%; 1/20) was only sensitive to azithromycin, gentamicin, meropenem and tobramycin.

### Pathogens identified and antimicrobial susceptibility pattern of gram-positive bacteria isolated from urine samples obtained from pregnant women

Slightly, more than half 56.8% (25/44) of asymptomatic bacteriuria cases were gram-positive bacteria, (Table 3). From these 25 cases of gram-positive bacteria, only four etiologies/agents were identified; coagulase negative *Staphylococcus* species (52%; 13/25) with the highest prevalence among the gram-positives followed by *Enterococcus* species (20%; 5/25), *S. aureus* (16%; 4/25) and *S. agalactiae* (8%; 2/25). The coagulase negative *Staphylococcus* species had 100% sensitivity to chloramphenicol, gentamicin and clindamycin. With 92.3% sensitivity to tetracycline, 23.1% sensitivity to ciprofloxacin, 84.6% sensitivity to oxacillin and trimethoprim-sulfamethoxazole and 69.2% sensitivity to azithromycin and erythromycin. All the *Enterococcus* species (100%) were sensitive to ofloxacin and ampicillin. All (100%) *S. aureus* were sensitive to erythromycin, azithromycin, chloramphenicol, ciprofloxacin, gentamicin, tetracycline, oxacillin and clindamycin. All the *S. agalactiae* (100%) showed sensitivity to erythromycin, chloramphenicol, oxacillin, clindamycin, ofloxacin and ampicillin.

**Table 3:** Pathogens identified and antimicrobial susceptibility patterns of gram-positive bacteria isolated from urine samples obtained from pregnant women.

Characteristic	Overall, N = 25^*1*^	CONS^*^, N = 13^*1*^	*Enterococcus spp*, N = 5^*1*^	*S. aureus*, N = 4^*1*^	*S. agalactiae*, N = 2^*1*^	*S. pneumoniae*, N = 1^*1*^
	n (%)	n (%)	n (%)	n (%)	n (%)	n (%)
Erythromycin						
*Intermediate*	5 (20)	0 (0)	5 (100)	0 (0)	0 (0)	0 (0)
*Resistant*	4 (16)	4 (31)	0 (0)	0 (0)	0 (0)	0 (0)
*Sensitive*	16 (64)	9 (69)	0 (0)	4 (100)	2 (100)	1 (100)
Azithromycin^**^ n=17						
*Intermediate*	0 (0)	0 (0)	0 (0)	0 (0)	0 (0)	0 (0)
*Resistant*	4 (24)	4 (31)	0 (0)	0 (0)	0 (0)	0 (0)
*Sensitive*	13 (76)	9 (69)	0 (0)	4 (100)	0 (0)	0 (0)
Chloramphenicol						
*Intermediate*	0 (0)	0 (0)	0 (0)	0 (0)	0 (0)	0 (0)
*Resistant*	5 (20)	0 (0)	5 (100)	0 (0)	0 (0)	0 (0)
*Sensitive*	20 (80)	13 (100)	0 (0)	4 (100)	2 (100)	1 (100)
Ciprofloxacin^**^ n=17						
*Intermediate*	1 (5.9)	1 (7.7)	0 (0)	0 (0)	0 (0)	0 (0)
*Resistant*	9 (53)	9 (69)	0 (0)	0 (0)	0 (0)	0 (0)
*Sensitive*	7 (41)	3 (23)	0 (0)	4 (100)	0 (0)	0 (0)
Gentamicin^**^ n=17						
*Intermediate*	0 (0)	0 (0)	0 (0)	0 (0)	0 (0)	0 (0)
*Resistant*	0 (0)	0 (0)	0 (0)	0 (0)	0 (0)	0 (0)
*Sensitive*	17(100)	13(100)	0 (0)	4 (100)	0 (0)	0 (0)
Tetracycline						
*Intermediate*	1 (4.0)	1 (7.7)	0 (0)	0 (0)	0 (0)	0 (0)
*Resistant*	7 (28)	0 (0)	5 (100)	0 (0)	2 (100)	0 (0)
*Sensitive*	17 (68)	12 (92)	0 (0)	4 (100)	0 (0)	1 (100)
Oxacillin ^**^ n=20						
*Intermediate*	0 (0)	0 (0)	0 (0)	0 (0)	0 (0)	0 (0)
*Resistant*	3 (15)	2 (15)	0 (0)	0 (0)	0 (0)	1 (100)
*Sensitive*	17 (85)	11 (85)	0 (0)	4 (100)	2 (100)	0 (0)
Trimethoprim-sulfamethoxazole						
*Intermediate*	0 (0)	0 (0)	0 (0)	0 (0)	0 (0)	0 (0)
*Resistant*	14 (56)	2 (15)	5 (100)	4 (100)	2 (100)	1 (100)
*Sensitive*	11 (44)	11 (85)	0 (0)	0 (0)	0 (0)	0 (0)
Clindamycin^**^ n=20						
*Intermediate*	0 (0)	0 (0)	0 (0)	0 (0)	0 (0)	0 (0)
*Resistant*	0 (0)	0 (0)	0 (0)	0 (0)	0 (0)	0 (0)
*Sensitive*	20 (100)	13 (100)	0 (0)	4 (100)	2 (100)	1 (100)
Ofloxacin^**^ n=8						
*Intermediate*	0 (0)	0 (0)	0 (0)	0 (0)	0 (0)	0 (0)
*Resistant*	0 (0)	0 (0)	0 (0)	0 (0)	0 (0)	0 (0)
*Sensitive*	8 (100)	0 (0)	5 (100)	0 (0)	2 (100)	1 (100)
Ampicillin^**^ n=8						
*Intermediate*	0 (0)	0 (0)	0 (0)	0 (0)	0 (0)	0 (0)
*Resistant*	0 (0)	0 (0)	0 (0)	0 (0)	0 (0)	0 (0)
*Sensitive*	8 (100)	0 (0)	5 (100)	0 (0)	2 (100)	1 (100)

^*1*^n (%):- n ~ Frequency, (%) ~ Percentages,

* coagulase negative staphylococci,

** some bacteria were not tested for the drug hence n not summing up to 25.

Interestingly, one pregnant woman had both gram-negative and gram-positive bacteria (*E. coli* and *S. agalactiae*).

### Risk factors associated with asymptomatic bacteriuria among pregnant women

A fixed logistic model (estimated using maximum likelihood [ML]) was fitted to predict associations of asymptomatic bacteriuria with previous history of urinary tract infection, level of education, occupation and maternal age. The effect of level of education [secondary level] was statistically significant and positive (AOR = 2.47, 95% CI [1.09, 5.98], p = 0.036). Meaning, women with at least secondary level of education were at 2.47 times higher odds of getting asymptomatic bacteriuria than primary level and below. There was statistically significant association between age and asymptomatic bacteriuria both at crude and adjusted model. The effect of maternal age [25–34 years] was statistically significant and positive (AOR = 2.23, 95% CI [1.07, 4.63], p = 0.030). Implying that women between 25-34 years of age were at 2.23 times higher odds of getting asymptomatic bacteriuria than their counterparts aged 15-24 years old. There was a negative association between previous history of Urinary tract infection and asymptomatic bacteriuria, though insignificant. The effect of previous history of urinary infection [yes] was statistically insignificant and negative (AOR = 0.56, 95% CI [0.29, 1.09], p = 0.086). Those who had previous history of urinary tract infection (UTI) had 44% reduction in ASB as compared to those with no history of urinary infection. The effect of occupation [working/employed] was statistically insignificant (AOR = 1.68, 95% CI [0.82, 3.64], p = 0.168). The odds of asymptomatic bacteriuria (ASB) was 68% high among the women who were either employed/working compare to their counter parts (Table 4).

**Table 4:** Risk factors associated with asymptomatic bacteriuria among pregnant women.

Characteristic	Crude Odds Ratio			Adjusted Odds Ratio		
**OR** ^*1*^	**95% CI** ^*1*^	**p-value**	**AOR** ^*1*^	**95% CI** ^*1*^	**p-value**
Previous history of UTI						
*No*	—	—		—	—	
*Yes*	0.58	0.31, 1.10	0.1	0.56	0.29, 1.09	0.086
Education level						
*Primary and below*	—	—		—	—	
*Secondary*	1.99	0.94, 4.51	0.083	2.47	1.09, 5.98	0.036
*Tertiary/university*	1.02	0.35, 2.84	>0.9	1.2	0.39, 3.51	0.7
Occupation						
*Unemployed/student*	—	—		—	—	
*Working/Employed*	1.74	0.88, 3.66	0.12	1.68	0.82, 3.64	0.2
Age						
*15-24*	—	—		—	—	
*25-34*	2.02	1.02, 3.99	0.041	2.23	1.07, 4.63	0.03
*35-44*	0.8	0.22, 2.25	0.7	1.13	0.30, 3.57	0.8

^*1*^OR = Odds Ratio, AOR = Adjusted Odds Ratio, CI = Confidence Interval

## Discussion

This study from western Kenya reports asymptomatic bacteriuria prevalence of 16.3%,which is lower than the 21.5% reported in Nairobi, Kenya [9], but similar to the 16.9% reported in Ethiopia [1], and higher than the 10% reported in India [16]. These differences may be attributed to geographical variations, differing genital hygiene practices among pregnant women, and health infrastructure of the different settings, socioeconomic status variations, and even difference in study designs [8].

Women aged 25-34 years had the highest bacteriuria (24.0%), comparable with findings from tertiary hospitals in India [17,18], probably due to high sexual activity of the child-bearing age, or increased parity, or gravidity [19]. In this study, asymptomatic bacteriuria was predominant in the 3^rd^ trimester (19.3%) followed by 1^st^ trimester (17.7%), and 2^nd^ trimester (12.0%), similar to other studies in Tanzania [2] and Nigeria [20], which found high prevalence of asymptomatic bacteriuria in 3^rd^ trimester. A study in west India [21] found lower (10.6%) prevalence in the first trimester, similar to another in Nairobi [9] which found that women in the first trimester had the lowest prevalence of asymptomatic bacteriuria. Although the latter in contrast found trimester 2 to have the highest prevalence of asymptomatic bacteriuria. This may imply that advanced pregnancy enhances vulnerability to urinary tract infections [2,13], mostly due to urethral dilation, which increases bladder volume, reducing urethral tone and aiding bacterial growth in urine [22]. This also explains why pregnant women who experience urinary tract infection or asymptomatic bacteriuria typically begins experiencing symptoms in the sixth week of pregnancy and peak between 22^nd^ and 24^th^ week [7,22]. The difference in the prevalence may also be due to the difference in sample size of the various studies, geographical differences, social habits in different communities and health related practices [2,13].

Among the cases positive for asymptomatic bacteriuria, one exhibited mixed growth indicative of co-infection [23], while the remaining 43 showed single bacterial colony growth. Gram-positive bacteria were more prevalent than gram-negative bacteria, similar to a report from Ethiopia [24]. The most frequent isolate in this study was *E. coli* (36.36%), followed by CONS (29.55%), similar to findings from India [22] and Nigeria [19]. Normally, *E. coli* is common in the perineum, hence poor personal hygiene can lead to infection, and its unique structure allows it to penetrate and proliferate within the uroepithelium, causing invasive infections [22]. Prior research has regarded CONS isolated from urine samples as contaminants, and did not assign them any importance [25], although a considerable number of CONS have in recent years been identified as causal agents for UTI [21]. Other organisms isolated in this study were gram-negative *K. pneumoniae, P. mirabilis, P. aeruginosa,* and gram-positive *Enterococcus species, S. aureus* and *S. agalactiae*, which largely agree with earlier reports from India [18,22].

All gram-negative bacteria were resistant to tetracycline and trimethoprim-sulfamethoxazole, except for *E. coli,* which showed resistance to nalidixic acid, ampicillin, and trimethoprim-sulfamethoxazole, but was sensitive to other tested drugs. These findings are consistent with those of a study in Ethiopia [8], but contrasts with a Nigerian report of high resistance among *E. coli* and *K. pneumoniae* to commonly used drugs, especially ceftriaxone [19]. This difference could be explained by variations in the frequency of antimicrobial usage, differences in the regulations governing antimicrobial use in different settings, and irrational use of antimicrobials, especially as self-medication [8].

K*. pneumoniae* were sensitive to most of the drugs tested except trimethoprim-sulfamethoxazole and tetracycline, and intermediate resistance to amoxicillin-clavulanic acid. The high sensitivity is comparable to reports from India that found high sensitivity to tobramycin and gentamicin [16,26]. The observed high resistance to tetracycline and trimethoprim-sulphamethoxazole can be attributed to the ease of access to these drugs over the counter, hence misuse [16].

*P. mirabilis* showed resistance to amoxicillin-clavulanic acid, ampicillin, chloramphenicol, tetracycline and trimethoprim-sulfamethoxazole, but intermediate for gentamicin, comparable with findings from India [22]. Part of the reason for this resistance is that *P. mirabilis* has the ability to develop biofilms, a known potent virulence factor [27]. *Proteus* species also produce urease, which hydrolyzes urea when it is present in excess in the urine, and promotes the formation of stones thus increasing the risk of pyelonephritis during pregnancy [8]. Certain *P. mirabilis* strains generate beta-lactamases, which degrade beta-lactam drugs like cephalosporin and penicillin, which are currently not effective against this bacterium [27]. The formation of biofilms and urease production is of great concern during pregnancy due to the body’s reduced immunity, hormonal effect on the urinary tract and increased risk of complications as a result of UTI [27].

The most resistance patterns were reported from *P. aeruginosa*, with resistance to amoxicillin-clavulanic acid, ampicillin, azithromycin, ceftriaxone, chloramphenicol, nalidixic acid, trimethoprim-sulfamethoxazole and tetracycline. It showed sensitivity to tobramycin, meropenem and gentamicin, but was intermediate for ciprofloxacin. These findings align with reports from Eastern Uganda [26]. Certain *P. aeruginosa* strains generate beta-lactamases, inactivating beta-lactam drugs like cephalosporin and penicillin, with some strains also being capable of forming biofilms [26].

The CONS showed high sensitivity to chloramphenicol, gentamicin, clindamycin, erythromycin, azithromycin, tetracycline, oxacillin and trimethoprim-sulfamethoxazole, but high resistance to ciprofloxacin, the latter being comparable to reports from Nairobi, where high resistance to fluoroquinolones was up to 18.8% [9]. All *Enterococcus* species were resistant to tetracycline, trimethoprim-sulfamethoxazole and chloramphenicol, but sensitive to ampicillin and ofloxacin. These findings are consistent with those from a teaching hospital in India that reported sensitivity to ampicillin among *Enterococcus* species [16], likely because it has less negative effects and is safer during pregnancy, so is likely used more frequently [18]. This could however vary with settings, due to variations in the sociodemographic and background features, variations in the degree of knowledge of UTI, and variations in antimicrobial usage [8].

All *S. aureus* were resistant to trimethoprim-sulfamethoxazole but sensitive to all the other tested drugs, while *S. agalactiae* showed resistance to tetracycline and trimethoprim-sulfamethoxazole but sensitivity to other tested drugs. This observed high resistance to trimethoprim-sulphamethoxazole in this study is consistent with findings from in Nairobi, the resistance likely due to easy drug accessibility, thus misuse [9]. The high resistance to commonly used drugs, especially ampicillin, azithromycin, ceftriaxone and amoxicillin-clavulanic acid among gram-negative bacteria is worth noting, and is likely driven by irrational use of these drugs, and their easy access over the counter in various outlets [18]. The widespread use of antibiotics, improper drug use and laxity in antibiotics monitoring are linked to multi-drug resistance development, which significantly affects patient outcomes, and undermines the effectiveness of a wide range of antibiotics [20,26]. Concerns have been expressed over the limited sensitivity to conventional antibiotics, suggesting that laws on the use of antibiotics should be strictly enforced, to control the rising antibiotic resistance, by limiting the amount of antibiotics exposed to these microorganisms [20,25].

Women with secondary or higher education were 2.47 times more likely to get asymptomatic bacteriuria than those with lower education, which agrees with a report from Ondo State Nigeria [2]. Ordinarily, though, it would be expected that women with higher levels of education practice proper hygiene to a greater extent than women with lower levels of education. These findings may thus be attributed to behavioral patterns, lifestyle factors, occupational factors such as stress or limited opportunities for hydration and frequent urination and higher utilization of public and communal amenities where infection could occur [2,28]. Women aged 25-34 years had 2.23 odds of getting asymptomatic urinary infections than those between 15-24 years of age, perhaps attributed to high sexual activity in this age group, which is comparable to findings from Nigeria [19] which identified the age group 20-29 years as having the highest prevalence of asymptomatic bacteriuria. Women with history of urinary tract infection had a 44% reduction in asymptomatic bacteriuria prevalence, possibly due to previous use of antibiotics hence the reduction in infection rate. The odds of asymptomatic bacteriuria among the employed was 68% higher compared to the unemployed, in part likely because of the shared public and communal amenities in their occupational setting [2].This study findings necessitates routine culture and susceptibility during antenatal care, tailored antimicrobial therapy based on the resistance data, enhanced screening protocols for the need for strengthened antimicrobial stewardship to prevent inherent use of antibiotic, a robust surveillance system to monitor antimicrobial resistance trends in order to guide proper clinical practices.

The study limitation was the cross-sectional nature of the sampling design and small sample size of 285. Given that participants may not remember past events or behaviors at a single point in time, this could result in recall bias. Generalizability to the larger population may be limited by the small sample size.

Consequently, the number of samples that tested positive for asymptomatic bacteriuria were few. Thus, adding a longitudinal component can help reduce recall bias by revealing causality and how things develop over time. Additionally, increasing the sample size may decrease random error and enhance representativeness.

## Conclusion

Asymptomatic bacteriuria was detected among pregnant women in this study, and both gram-positive and gram-negative bacteria were isolated, the predominant bacteria being *E. coli* and coagulase negative *Staphylococcus* species. Most isolates in this study were susceptible to the antibiotics used, although high resistance to antibiotics was observed among gram-negative bacteria. Great resistance to trimethoprim-sulfamethoxazole should be highly noted since it is a combination antibiotic used to treat bacterial infection. Finally, maternal age and educational level were found to be significantly associated with asymptomatic bacteriuria.

There is a need for routine screening and a more refined antimicrobial susceptibility testing, which is important for guiding treatment protocols

## References

[1] Wabe YA, Reda DY, Abreham ET, Gobene DB, Ali MM. Prevalence of Asymptomatic Bacteriuria, Associated Factors and Antimicrobial Susceptibility Profile of Bacteria Among Pregnant Women Attending Saint Paul’s Hospital Millennium Medical College, Addis Ababa, Ethiopia. Ther Clin Risk Manag. 2020;16:923–32. doi: 10.2147/TCRM.S267101 33061397 PMC7532909

[2] Onemu S, Ige, R, Onemu-Metitiri M, Uyigue P, Obeagu E. The prevalence of asymptomatic bacteriuria in pregnant women in Akure, Ondo State, Nigeria. DYSONA - Life Science. 2024;5:8.

[3] Adenike Fowora M, Adewale Osuolale K, Ogunsanya J, Uloma Onyeaghasiri F, Olaide Edu-Muyideen I, Akintunde G, et al. Prevalence and Risk Factors of Asymptomatic Bacteriuria Among Apparently Healthy Women in Lagos, Nigeria. WJPH. 2021;6(4):5. doi: 10.11648/j.wjph.20210604.20

[4] Azami M, Jaafari Z, Masoumi M, Shohani M, Badfar G, Mahmudi L, et al. The etiology and prevalence of urinary tract infection and asymptomatic bacteriuria in pregnant women in Iran: a systematic review and Meta-analysis. BMC Urol. 2019;19(1):43. doi: 10.1186/s12894-019-0454-8 31146773 PMC6543660

[5] Awoke N, Tekalign T, Teshome M, Lolaso T, Dendir G, Obsa MS. Bacterial Profile and asymptomatic bacteriuria among pregnant women in Africa: A systematic review and meta analysis. EClinicalMedicine. 2021;37:100952. doi: 10.1016/j.eclinm.2021.100952 34386744 PMC8343252

[6] Abu D, Abula T, Zewdu T, Berhanu M, Sahilu T. Asymptomatic Bacteriuria, antimicrobial susceptibility pattern and associated risk factors among pregnant women attending antenatal care in Assosa General Hospital, Western Ethiopia. BMC Microbiol. 2021;21(1):348. doi: 10.1186/s12866-021-02417-6 34915840 PMC8675524

[7] Farazi A, Jabbariasl M. Asymptomatic bacteriuria in pregnancy in the central region of Iran: Frequency, risk factors, and causative organisms. Clinical Epidemiology and Global Health. 2019;7(3):309–12. doi: 10.1016/j.cegh.2018.09.009

[8] Bizuwork K, Alemayehu H, Medhin G, Amogne W, Eguale T. Asymptomatic Bacteriuria among Pregnant Women in Addis Ababa, Ethiopia: Prevalence, Causal Agents, and Their Antimicrobial Susceptibility. Int J Microbiol. 2021;2021:8418043. doi: 10.1155/2021/8418043 34335781 PMC8313335

[9] Ayoyi AO, Kikuvi G, Bii C, Kariuki S. Prevalence, aetiology and antibiotic sensitivity profile of asymptomatic bacteriuria isolates from pregnant women in selected antenatal clinic from Nairobi, Kenya. Pan Afr Med J. 2017;26:41. doi: 10.11604/pamj.2017.26.41.10975 28451019 PMC5398259

[10] Alenazi A, aher I, Taha A, Elawamy W, Alshlash A, El-masry E. Pregnancy-associated asymptomatic bacteriuria and antibiotic resistance in the Maternity and Children’s Hospital, Arar, Saudi Arabia. J Infect Dev Ctries. 2023;17(12). 10.3855/jidc.18184 38252724

[11] Cotton E, Geraghty R, Umranikar S, Saeed K, Somani B. Prevalence of asymptomatic bacteriuria among pregnant women and changes in antibiotic resistance: a 6-year retrospective study. Journal of Clinical Urology. 2024;17(1):7. doi: insert_doi_here

[12] Garnizov T. Asymptomatic bacteriuria in pregnancy from the perspective of public health and maternal health care: review and case report. Biotechnology & Biotechnological Equipment. 2016;30(3):5.

[13] Sivalingarajah R, Balasingham B, Camilas JC. Asymptomatic bacteriuria in pregnancy. Sri Lanka J Obstet & Gynae. 2023;45(1):21–6. doi: 10.4038/sljog.v45i1.8091

[14] Alhamadani Y, Oudah A. Study of the bacterial sensitivity to different antibiotics which are isolated from patients with UTI using Kirby-Bauer method. Journal of Biomedicine and Biochemistry. 2022;1(2):5.

[15] Shoaib M, Satti L, Hussain A, Khursheed N, Sarwar S, Shah AH. Disc diffusion testing of azithromycin against clinical isolates of typhoidal salmonellae: A diagnostic conundrum. Cureus. 2021;13(7):e16777. 34513384 10.7759/cureus.16777 PMC8404649

[16] Talukdar B, Kalita D, Deka S, Mahela S. Prevalence and its antibacterial susceptibility pattern of asymptomatic bacteriuria in pregnancy of a teaching hospital. obgyn. 2023;9(2):216–20. doi: 10.21276/obgyn.2023.9.2.5

[17] Rohini U, Reddy G, Kandati J, Ponugoti M. Prevalence and associate risk factors of asymptomatic bacteriuria in pregnancy with bacterial pathogens and their antimicrobial susceptibility in a tertiary care hospital. Int J Reprod Contracept Obstet Gynecol. 2017;6(6):5.

[18] Totadhri M, Lakshmanan A, Saraswathy MP, Mane MS. Asymptomatic bacteriuria of pregnant women in a tertiary care centre. J Educ Health Promot. 2022;11:249. doi: 10.4103/jehp.jehp_1752_21 36325203 PMC9621380

[19] Kalgo Z, Yusuf A, Umar S, Mohammed B, Aliyu B, Gulumbe B. Prevalence of asymptomatic bacteriuria among pregnant women in Kebbi State, Nigeria. International Journal of Women’s Health Care. 2022;7(3):6.

[20] Mwei MK, Mchome B, John B, Maro E. Asymptomatic bacteriuria among pregnant women attending antenatal care at Kilimanjaro Christian Medical Centre in Northern Tanzania. Tanzania J Hlth Res. 2018;20(4). doi: 10.4314/thrb.v20i4.8

[21] Patel P, Patel M, Desai K. Prevalence of Asymptomatic Bacteriuria Among Pregnant Women Attending a Tertiary Care Hospital in Western India. Natl J Community Med. 2022;13(10):728–32. doi: 10.55489/njcm.131020222444

[22] Sonkar N, Banerjee M, Gupta S, Ahmad A. Asymptomatic bacteriuria among pregnant women attending tertiary care hospital in Lucknow, India. Dubai Med J. 2021;4(1):8.

[23] Sgayer I, Shamalov G, Assi S, Glikman D, Lowenstein L, Frank Wolf M. Bacteriology and clinical outcomes of urine mixed bacterial growth in pregnancy. Int Urogynecol J. 2024;35(2):347–53. doi: 10.1007/s00192-023-05672-5 37938399

[24] Ali IE, Gebrecherkos T, Gizachew M, Menberu MA. Asymptomatic bacteriuria and antimicrobial susceptibility pattern of the isolates among pregnant women attending Dessie referral hospital, Northeast Ethiopia: A hospital-based cross-sectional study. Turk J Urol. 2018;44(3):251–60. doi: 10.5152/tud.2018.07741 29733799 PMC5937645

[25] Gajdács M, Ábrók M, Lázár A, Burián K. Increasing relevance of Gram-positive cocci in urinary tract infections: a 10-year analysis of their prevalence and resistance trends. Sci Rep. 2020;10(1):17658. doi: 10.1038/s41598-020-74834-y 33077890 PMC7573585

[26] Nteziyaremye J, Iramiot SJ, Nekaka R, Musaba MW, Wandabwa J, Kisegerwa E, et al. Asymptomatic bacteriuria among pregnant women attending antenatal care at Mbale Hospital, Eastern Uganda. PLoS One. 2020;15(3):e0230523. doi: 10.1371/journal.pone.0230523 32191758 PMC7082119

[27] Armbruster CE, Mobley HLT, Pearson MM. Pathogenesis of Proteus mirabilis Infection. EcoSal Plus. 2018;8(1):10.1128/ecosalplus.ESP-0009–2017. doi: 10.1128/ecosalplus.ESP-0009-2017 29424333 PMC5880328

[28] Sujatha R, Nawani M. Prevalence of asymptomatic bacteriuria and its antibacterial susceptibility pattern among pregnant women attending the antenatal clinic at kanpur, India. J Clin Diagn Res. 2014;8(4):DC01-3. doi: 10.7860/JCDR/2014/6599.4205 24959438 PMC4064844

